# Potential role of polycyclic aromatic hydrocarbons in air pollution-induced non-malignant respiratory diseases

**DOI:** 10.1186/s12931-020-01563-1

**Published:** 2020-11-13

**Authors:** Marit Låg, Johan Øvrevik, Magne Refsnes, Jørn A. Holme

**Affiliations:** 1 Section of Air Pollution and Noise, Department of Environmental Health, Norwegian Institute of Public Health, Skøyen, PO Box 222, 0213 Oslo, Norway; 2 Department of Biosciences, Faculty of Mathematics and Natural Sciences, University of Oslo, Oslo, Norway

**Keywords:** PAHs, Asthma, Obstructive lung diseases, Inflammation, ROS, AhR

## Abstract

Epidemiological studies have found strong associations between air pollution and respiratory effects including development and/or exacerbation of asthma and chronic obstructive pulmonary disease (COPD) as well as increased occurrence of respiratory infections and lung cancer. It has become increasingly clear that also polycyclic aromatic hydrocarbons (PAHs) may affect processes linked to non-malignant diseases in the airways. The aim of the present paper was to review epidemiological studies on associations between gas phase and particle-bound PAHs in ambient air and non-malignant respiratory diseases or closely related physiological processes, to assess whether PAH-exposure may explain some of the effects associated with air pollution. Based on experimental in vivo and in vitro studies, we also explore possible mechanisms for how different PAHs may contribute to such events. Epidemiological studies show strongest evidence for an association between PAHs and asthma development and respiratory function in children. This is supported by studies on prenatal and postnatal exposure. Exposure to PAHs in adults seems to be linked to respiratory functions, exacerbation of asthma and increased morbidity/mortality of obstructive lung diseases. However, available studies are few and weak. Notably, the PAHs measured in plasma/urine also represent other exposure routes than inhalation. Furthermore, the role of PAHs measured in air is difficult to disentangle from that of other air pollution components originating from combustion processes. Experimental studies show that PAHs may trigger various processes linked to non-malignant respiratory diseases. Physiological- and pathological responses include redox imbalance, oxidative stress, inflammation both from the innate and adaptive immune systems, smooth muscle constriction, epithelial- and endothelial dysfunction and dysregulated lung development. Such biological responses may at the molecular level be initiated by PAH-binding to the aryl hydrocarbon receptor (AhR), but possibly also through interactions with beta-adrenergic receptors. In addition, reactive PAH metabolites or reactive oxygen species (ROS) may interfere directly with ion transporters and enzymes involved in signal transduction. Overall, the reviewed literature shows that respiratory effects of PAH-exposure in ambient air may extend beyond lung cancer. The relative importance of the specific PAHs ability to induce disease may differ between the biological endpoint in question.

## Background

Air pollution is among the leading environmental health risk factors, estimated to cause between four and nine million deaths globally [1, 2]. Some of the strongest associations have been reported between fine particular matter (PM_2.5_) and development or exacerbation of adverse respiratory outcomes, including asthma, chronic obstructive pulmonary disease (COPD), respiratory infections and lung cancer [2–6]. A causal link between PM and several of the diseases has been established with support from experimental studies in humans, animals and cells [2, 3, 5, 6]. The mechanisms through which PM causes respiratory effects are multifactorial and still discussed [7–9]. Although it has become increasingly clear that the chemical composition of particles is of importance for many of their biological and toxicological effects [10–13], the precise mechanisms involved may vary with the biological endpoint studied [7, 14].

Incomplete combustion of coal and various organic materials as fossil fuels and cigarette smoking produces a mixture of pollutants including PM. PM typically consists of a carbon core with mixtures of organic chemicals adhered to the surface [15–17]. The specific composition and amount of organic chemicals are highly dependent on fuel burned and combustion technology. The levels of organic chemicals are often found to be in the range 20–30% of total particle mass, but may reach as much as 90% [18, 19].

Of the organic chemicals in PM, some of the polycyclic aromatic hydrocarbons (PAHs) are considered to have the highest toxic potential in terms of cytotoxicity, mutagenicity and carcinogenicity [20]. High-molecular weight PAHs containing five or more aromatic rings are mainly found bound to PM, while smaller PAHs containing four or less aromatic rings are found to a greater extent in the gas phase [20, 21]. However, due to their higher total concentrations in the air when compared to larger PAHs, low-molecular PAHs nevertheless tend to be the dominating PAHs bound to PM. Levels of phenanthrene and pyrene bound to diesel exhaust and wood smoke particles typically exceed the level of the carcinogenic indicator benzo[*a*]pyrene (B[*a*]P) by orders of magnitude [20]. The relative amount of PAHs adhered to the PM depend on source, but is to a large degree also affected by temperature. Thus, PAHs levels attached to PM during winter may be tenfold higher compared to summer when the more volatile PAHs to a greater extent evaporate and exist in gas phase [22]. Although indoor sources also contribute to the total air-borne PAH exposure [23–25], indoor air is often influenced by outdoor sources [26].

PAHs encompass hundreds of chemicals with two or more fused aromatic rings. Based partly on occurrence and partly on toxicity, special attention has been given to the 16 PAHs classified as priority pollutants (USEPA, 2005): naphthalene, acenaphthylene, acenaphthene, fluorene, phenanthrene, anthracene, fluoranthene, pyrene, benz(a)anthracene, chrysene, benzo(b)fluoranthene, benzo(k)fluoranthene, B[*a*]P, dibenz(a,h)anthracene, benzo(ghi)perylene and indeno(1,2,3-cd)pyrene. Metabolite levels in blood and urine of low molecular weight PAHs such as pyrene, phenanthrene and naphthalene are often used as surrogates to assess airborne PAH–exposure [27, 28]. This is due to the fact that these PAHs to a lesser degree than larger ones are found in food. PAHs absorbed from food, a major source of the larger PAHs [21], will be metabolized in the liver by first path elimination. Thus, inhalation by exposure is considered more important for respiratory effects of PAHs.

The toxicity of PAHs is linked to the formation of reactive electrophilic metabolites and/or activation of cellular receptors including the aryl hydrocarbon receptor (AhR) [29–32]. Health effects of PAH exposure via inhalation have predominately been linked to their carcinogenic potential [20]. Several of the PAHs are potent animal carcinogens [33]. Accordingly, risk assessments of PAHs are based on toxicity equivalency factors (TEFs) calculated as their relative carcinogenicity compared to B[*a*]P, and current air quality guidelines are limited to the carcinogenic effects of B[*a*]P [34]. This review indicates PAHs may also affect processes linked to non-malignant respiratory diseases, and both the types of PAH involved and mechanisms by which they exert their effects, may be different from those inducing cancer. Hence the use of TEF-factors, may not necessarily be equally relevant for non-carcinogenic outcomes following PAH exposure.

The aim of the present paper was therefore to review epidemiological studies on associations between PAHs in ambient air pollution and non-malignant respiratory diseases or processes linked to the diseases. Furthermore, based on experimental animal and in vitro studies, we also explore possible mechanisms for how the various PAH may be contributing factors to such events.

## Search strategy

The literature search was performed in two steps. The first strategy was to use keywords in PubMed, called MeSH-terms (Medical Subject Headings) and the following search terms was selected; ((((((("Air Pollutants"[Mesh]) OR "Air Pollution"[Mesh]) OR "Environmental Exposure"[Mesh]) OR "Inhalation Exposure/adverse effects"[Mesh])) AND "Polycyclic Aromatic Hydrocarbons"[Mesh])) AND ((((("Respiratory Tract Diseases"[Mesh]) OR "Respiratory Function Tests"[Mesh]) OR "Respiratory Physiological Phenomena"[Mesh])) NOT "Neoplasms"[Mesh]) (29.5.2018). All the 89 publications identified were screened at abstract level. In total 24 relevant epidemiological studies exploring non-malignant respiratory diseases exposure to environmental levels of PAHs were included; three occupational studies at high PAHs levels were also included as proof of principle. Six papers were identified by tracking the citation network (cited and citing papers) of identified papers. Additional search was also done for PAHs in indoor air, and three of these studies were added. Importantly, personal air samplers have been used in many of the epidemiological studies implying that both indoor and outdoor sources contributed to the measured values.

The first literature search focused on air pollution and epidemiological studies. However, additional strategies were used to identify important experimental and mechanistic studies on PAHs, since they may support the epidemiological studies and explain the biological plausibility. Thus, several literature searches were performed in PubMed using various combinations of PAHs, specific PAHs or AhR and terms linked to respiratory diseases, including inflammatory responses, immune system, autonomic nervous system, lung development, lung infection, asthma and COPDs. The number of publications identified were very high, thus first rapidly screened for relevance at the title and next at abstract level. Additional papers were found by tracking citations of identified papers. Relevant information from several hundred papers was evaluated. We highlight research suggesting and elucidating how PAHs and AhR-dependent mechanisms might be linked to cellular processes central in development and exacerbation of respiratory diseases.

## PAH exposure and non-malignant respiratory outcomes: evidence from epidemiological studies

Air pollution is associated with the development and exacerbation of several non-malignant respiratory diseases [2, 3, 5, 6]. Importantly, exposure to PAHs has been associated with many of the same respiratory effects, including asthma and impaired respiratory functions, obstructive lung diseases and increased risk of respiratory infections (Table 1).

**Table 1:** The epidemiological studies included in the review

Study design	Population	Location	Exposure characterization	Health end-points	Correlation/findings	References
Case–control	195 children up to 15 years (98 asthma pediatric patients and 97 healthy controls)	Arabic children	Serum concentrations: Naphthalene, 4H-cyclopenta[def]phenanthrene, 1,2-benzanthracene, chrysene, benzo(*e*)acephenanthrylene, pyrene, B[*a*]P, anthracene, fluorene, phenanthrene, fluoranthrene, benzo(*e*)pyrene	Asthma (IgE, resistin, GMCSF, IFN-γ, IL-4, IL-5, CXCL8 and IL-10)	Naphthalene, 4H-cyclopenta[def]phenanthrene, 1,2-benzanthracene, chrysene, benzo(*e*)acephenanthrylene associated with IgE, restin, GMCSF, IFN-γ, IL-4, IL-5, CXCL8 and IL-10	[52]
Case–control	453 kindergarten children (126 asthmatic children and 327 controls)	From a cohort recruited in 2010 in Taipei, China	Urine concentrations: 1- hydroxy-pyrene and 8-OHdG	Information about asthma Total IgE	1- hydroxy-pyrene significantly associated with asthma (OR 1.42) and IgE	[53]
Case–control	42 asthmatic children 20 healthy controls	Hospital-based study in Lucknow, northern India	Blood levels: naphthalene, acenapthene, phenanthrene, anthracene, fluoranthene, pyrene, benzo (b) fluo., benzo (k) fluo., benzo (a) pyrene, di benz (a,h) anthracene	Blood levels of antioxidants (catalase, superoxide dismutase, malon-dialdehyde, reduced GSH)	Blood levels of phenanthrene were significantly higher in asthmatics than in healthy children. Blood GSH level was also associated with asthma	[54]
Case–control	507 asthmatic adults 536 matched controls	The asthma cases were recruited during 2010–2012 from a hospital in Wuhan, China	Urine concentrations: 1-hydroxynaphthalene (1-OHNAP), 2-OHNAP, 9-hydroxyfluorene (9-OHFLU), 2-OHFLU, 4-hydroxyphenanthrene (4-OHPHE), 9-OHPHE, 3-OHPHE, 1-OHPHE, 2-OHPHE, 1-Hydroxypyrene, 6-hydroxychrysene and 3-hydroxybenzo[a]pyrene ∑OH-PAHs 12 PAHs metabolites	Increased risk of adult asthma diagnosed by physicians	Each 1-unit-increase in natural log-transformed concentrations of 2-hydroxyfluorene (2-OHFLU), 4- hydroxyphenanthrene (4-OHPHE), 1-OHPHE, 2-OHPHE, 1-Hydroxypyrene (1-OHPYR) and ∑OH-PAHs were significantly associated with elevated risk of adult asthma with ORs of 2.04, 2.38, 2.04, 1.26, 2.35 and 1.34, respectively	[63]
Panel study	72 children with asthma (7–12 years)	Area with heavy industry, Montreal, Canada	Personal monitoring of various air pollutants including PAHs. Median personal concentration of total PAHs was 130 µg/m^3^	Respiratory function (spirometry; FEV_1_, FVC)	A possible small decrease in respiratory function with total concentration of PAHs	[62]
Panel study	560 adults of 60 years or older	Seoul, Korea	Urine concentrations: 1-hydroxy-pyrene 2-naphthol	Lung function tests (spirometry) Genotyping of *CYP1A1*	Urinary 1-hydroxy-pyrene levels were inversely associated with FEV_1_/FVC Haplotype-based *CYP1A1* polymorphism modified the risk	[66]
Panel study	88 adult patients with chronic cough	Kanazawa University Hospital, Ishikawa Perfecture, Japan	Ambient air monitoring of six PAH compounds including fluoranthene, pyrene, chrysene, benzo[b]fluoranthene, benzo[k]fluoranthene, benzo[a]pyrene	Diary of cough and symptoms Spesific IgE Exhaled NO	Association between ambient PAH and cough occurrence. The non-asthma group had slightly higher OR for cough per 1 ng/m^3^ PAHs than the asthmatics	[69]
Cross-sectional	467 children with and without asthma followed up to 8 years	Fresno, CA, USA	Used a spatiotemporal model to estimated individual exposure: $\sum$ PAHs with 4, 5, and 6 rings (PAHs456)	Respiratory function (spirometry; FEV_1_, FEF_25-75_)	Non-asthmatics: $\sum$ PAHs456 exposure during previous periods (3–12 months) associated with decrease in FEV_1_ Asthmatics: no association	[60]
Cross-sectional	64 schoolchildren	Mexico city, Mexico	Urine concentration: Monohydroxy-PAHs	Respiratory function (spirometry; FEV_1_, FVC) pH of exhaled breath condensate (EBC)	Increase of 2-hydroxy-fluorene was significantly negatively associated with FEV_1_, FVC and pH of EBC	[61]
Cross-sectional	3531 people (non-smoker) from 6 to 79 years	A Canadian population	Urinary concentrations: 1-/2-hydroxy-napthalene, 2-/3-/9-hydroxy-fluorene and 1-/2-/3-/4-/9-hydroxy-phenanthrene, 1-hydroxy-pyrene Total 11 PAHs	Respiratory function (spirometry; FEV_1_ and FVC)	8 PAH metabolites (2-hydroxy-napthalene, 1-/2-hydroxy-phenanthrene, 2-/ 3-/9-hydroxy-fluorene and 3-/4-hydroxy-phenanthrene) were associated with decrements of FEV_1_ and FVC	[65]
Birth cohort	333 newborns from non-smoking women (aged 18–35)	Krakow, Poland	Personal monitoring of PAHs inhalation in pregnant women for a 48 h period: ∑ PAHs (benzo(a)anthracene, benzo(b)fluoranthene, benzo(k)fluoranthene, benzo(g,h,i)perylene, benzo(a)pyrene,chrysene/ iso-chrysene, dibenzo(a,h)-anthracene, indeno(1,2,3-c,d)pyrene, and pyrene)	Respiratory symptoms based on interview of the mothers	Prenatal PAH exposure associated significantly with occurrence of respiratory outcomes: ear infections, cough, throat infections, observed in infants over the first year of life	[42]
Birth cohort	257 newborns from non-smoking women (aged 18–35)	Krakow, Poland	Personal monitoring of PAHs and PM_2.5_ inhalation in pregnant women for a 48 h period. In addition indoor and outdoor residential air levels of PAHs (both particle-bound and gaseous) and particle mass were measured	Number of wheezing days based on interview of the mothers. The new-borns were followed-up every 3 or 6 months with 12 health visits	Prenatal and postnatal exposure to PAHs were associated positively with the severity of wheezing days and recurrent wheezing	[43]
Birth cohort	339 newborns from non-smoking women (aged 18–35)	Krakow, Poland	Personal monitoring of PM_2.5_ inhalation in pregnant women for a 48 h period. BaP-DNA adducts in umbilical cord blood	Incidence rate ratio for the number of wheezing days	Prenatal levels of BaP-DNA adducts and prenatal PM_2.5_ levels associated positively with the number of wheezing days during the first 2 years of life	[44]
Birth cohort	439 newborns from non-smoking women (aged 18–35)	Krakow, Poland	Personal monitoring of PAH inhalation in pregnant women during the second trimester Barbecued meat consumption	Birth outcomes (birth weight, length, head circumference)	Airborne PAH and consumption barbecued meat associated with deficit in birth weight	[41]
Birth cohort	195 non-asthmatic children of non-smoking mothers	Krakow, Poland	Personal air monitoring of PAH inhalation in pregnant women for a 48 h period. Geometric mean of PAH concentrations was 20.1 ng/m^3^. For each child residential air born PAH indoor (21.3 ng/m^3^) and outdoor (32.5 ng/m^3^) monitoring was conducted at age of 3	Respiratory function (spirometry; FVC, FEV_05_, FEV_1_, FEF_25-75_) at age 5–9	Prenatal PAH exposure association with reduction of FEV1, FEF_25-75_. Also postnatal residential indoor PAH levels were associated with reduced FEV_1_ and FEF_25-75_	[48]
Birth cohort	222 children age 5 years living in inner-city	New York, USA	Urine concentrations: 10 monohydroxy-PAHs detected out of 24 metabolites measured	Questionnaires to the mothers: Child’s medical history, respiratory symptoms and health-care utilization Total and specific IgE	Increased 3- hydroxyfluorene and –phenanthrene associated with higher anti-mouse IgE. Also other PAH metabolites showed association using different analyses No association between PAH metabolites and respiratory symptoms	[55]
Birth cohort	Children from 727 non-smoking, African American or Dominican women, the ages 18–35, living in inner-city	New York, USA	Personal monitoring of 8 non-volatile PAHs and pyrene in air for 48 h during the third trimester of pregnancy Dust collected from homes at different time points both pre- and post-natal for allergen determination	Allergen specific IgE Glutathione-S-µ1 (GSTM1) gene polymorphisms	Prenatal exposure to non-volatile PAHs and cockroach allergen were associated with increased risk of allergic sensitization. Children null for GSTM1 mutation most vulnerable	[46]
Birth cohort	~ 700 children living in inner-city	New York, USA	Personal monitoring of 8 non-volatile carcinogenic PAHs and pyrene in air for 48 h during the third trimester of pregnancy	Parental report on asthma symptoms in children prior to age of 5 Methylation sensitive restriction fingerprinting of DNA from umbilical cord white blood cells of some cohort children	Maternal ∑ PAH exposure exceeding 2.41 ng/m^3^ was significantly associated with methylation of a specific DNA sequence (*ACSL3*) and with the parental report of asthma symptoms in children prior to age 5	[45]
Birth cohort	Children from 303 non-smoking women living in inner-city	New York, USA	Personal monitoring of 8 carcinogenic PAHs in air for 48 h during the third trimester of pregnancy	Questions to the mothers: Child’s medical history, respiratory symptoms and health-care utilization	Prenatal exposure to PAH and early exposure to environmental tobacco smoke (ETS) was associated with increased respiratory symptoms and probable asthma by age 12 to 24 months	[49]
Birth cohort	Children from 725 non-smoking healthy women living in inner-city	New York, USA	Personal air monitoring of 8 non-volatile carcinogenic PAHs and pyrene in air for 48 h during the third trimester of pregnancy Prenatal and postnatal ETS were defined as the report of any smokers in the home. Plasma cotinine was measured in cord blood and child blood	Questionnaires on doctor diagnosis of asthma, emergency room visits due to breathing problems and use of asthma medications in the past 12 month at ages of 5 and 6 years. Total and specific IgE	Combined prenatal PAH and ETS exposure were associated with asthma, but not IgE. Prenatal PAH exposure alone was neither associated with asthma nor IgE at age 5 to 6 years	[50]
Birth cohort	Children from 354 non-smoking healthy women living in inner-city	New York, USA	Personal air monitoring of 8 non-volatile carcinogenic PAHs and pyrene in air for 48 h during the third trimester of pregnancy PAH exposure at 5 to 6 years of age was measured from 2-week residential indoor monitoring	Questionnaires on wheeze in the past 12 months, physician diagnosis of asthma and asthma medication in the past 12 month at ages 5 and 6 years. Emergency room visits due to breathing problems Total and specific IgE	Repeated high exposure to pyrene was associated with asthma, medication use and emergency room visits. No associations between the levels of the 8 non-volatile carcinogenic PAHs and asthma were observed. Non-atopic children seem more susceptible to respiratory consequences of early pyrene exposure	[51]
Birth cohort	455 mothers and their children	Lodz district, Poland	Urine concentration: 1-hydroxy-pyrene (1-HP)	Interview-based; The children’s health status was assessed at the age of 10–18 months and at 2 years	Higher 1-HP in mothers at 20–24 weeks of pregnancy increased the risk of respiratory infections in children during their first year of life. Higher 1-HP in children increased their risk of food allergy	[78]
Birth cohort	3378 births in a polluted district 1505 in a control district	Two Czech districts: Teplice with high air pollution and Prachatice with lower exposure	Air monitoring of mean PM_10_, PM_2.5_ and B[*a*]P estimation for each mother	Pregnancy outcomes from medical records; intrauterine growth retardation, respiratory morbidity up to 10 years of age DNA adducts, micronuclei and gene expression profiles in cord blood	PM_10_ and B[*a*]P exposure in the first month of gestation were associated with intrauterine growth retardation. Increased concentrations of PM_2.5_ and B[a]P associated with development of bronchitis in preschool children DNA adducts and micronuclei were elevated in cord blood from births in high polluted area	[40]
Birth cohort with repeated-measures	1133 children born in 1994–1998 followed to 3 or 4.5 years of age	Two Czech districts: Teplice with high air pollution and Prachatice with lower exposure	Air monitoring of PM_2.5_ and 12 PAHs (gas and particle phases)	Questionnaires and medical records: Respiratory illnesses; bronchitis and total lower respiratory illnesses	Rising pollutants concentrations (ambient PAHs and PM_2.5_) were associated with increased bronchitis rates. Associations were stronger for longer pollutant-averaging periods, and among children > 2 years of age for PAH compared with fine particles	[79]
Cohort study	315 children aged 6–11 years with asthma followed from 2000 to 2008	Fresno, CA, USA	Ambient pollutant concentrations were collected from a central site and at selected homes. Measurements of PAHs: phenanthrene and the sum of nine selected PAH456, which includes fluoranthene, benz[a]anthracene, chrysene, benzo[*a*]pyrene, benzo[b]fluoranthene, benzo[k]fluoranthene, benzo[ghi]perylene, indeno[1,2,3-cd]pyrene, and dibenz[a,h]anthracene	Questionnaires and medical records: Increased wheeze	PAH exposure were associated with increased wheeze. The trend for increased wheeze persisted among all PAHs measured	[56]
Cohort study	2747 participants (18–80 years)	Wuhan, China	Urinary concentration: 1-hydroxy-naphthalene; 2-hydroxy-naphthalene; 2-hydroxy-fluorene; 9-hydroxy-fluorene 1-hydroxy-phenanthrene; 2-hydroxy-phenanthrene; 3-hydroxy-phenanthrene; 4-hydroxy-phenanthrene; 9-hydroxy-phenanthrene; 1-hydroxy-pyrene; 6-hydroxy-chrysene; and 3-hydroxy-benzo[a]pyrene	Respiratory function (spirometry; FEV_1_ and FVC)	Total and specific urinary PAH metabolites were associated with reduction of FEV_1_ and FVC	[67]
Cohort study	2739 participants (18–80 years)	Wuhan, China	Urinary concentrations: 12 mono-hydroxy-PAHs	Questionnaire Respiratory function (spirometry; FEV_1_ and FVC)	Urinary hydroxy-PAHs levels were marginally negatively related to FEV_1_. Low levels of education affected FEV_1_/FVC together with high exposure to PAHs	[68]
Cross-sectional	137 diesel engine testing workers (male) 127 controls	Workers at a diesel engine manufacturing plant in China	Airborne concentrations of 16 PAHs from PMs Urinary concentrations: 6 mono-hydroxy-PAHs	Respiratory function (spirometry)	Increasing levels of PAH metabolites were associated with decreases in respiratory function	[71]
Cohort study	1243 coke-oven workers	Coke-oven plant in Wuhan, China	Urinary concentrations: 12 mono-hydroxy-PAHs	Respiratory function (spirometry; FEV_1_ and FVC)	Total and specific urinary hydroxy-PAHs were associated with accelerated decline in FEV_1_/FVC	[70]
Cohort study	58,862 asphalt workers (men) employed between 1913 and 1999 36,831 persons in a subcohort never exposed to coal tar	Workers from Denmark, Finland, France, Germany Israel, the Netherlands, Norway	Estimation of exposure to bitumen fume, coal tar, B[*a*]P (marker for 4–6-ring PAHs), diesel exhaust, respirable silica and asbestos	Mortality from obstructive lung diseases	Estimated cumulative and average exposures to PAH and coal tar were associated with mortality from obstructive lung diseases	(72)

### Asthma and respiratory functions

Asthma is a chronic and heterogeneous disease characterized by recurrent airway obstruction, bronchial hyper-responsiveness and airway inflammation affecting both children and adults [35]. The number of people having asthma seems to remain at high prevalence, and it has been estimated that asthma is now affecting one of eight children worldwide [36]. Clinical and epidemiological studies have found that ambient pollution induces acute asthma exacerbation, and that the exposure also is associated with the onset of asthma [37, 38]. It should be emphasized that the etiology of asthma is complex, and the combination of genetic and other environmental factors including viral infections and allergens is likely to be involved.

*Developing effects from pre- and postnatal exposure.* PAHs have been found to affect placental functions [39]. Lipophilic PAHs can easily cross the placental barrier and exposure has been associated with alterations including reduced foetal growth and developmental toxicity [39]. A study in a region of the Czech Republic with high ambient concentrations of PAHs showed an association between prenatal PAH exposure and intrauterine growth retardation and higher respiratory morbidity [40]. Also in a study from a birth cohort in Krakow, Poland, airborne PAHs were associated with reduced birth weight [41]. However, it should be noted that the mothers’ circulating PAH-levels, which may reach the foetus, originate from both inhalation and diet.

There are several studies indicating that in utero exposure of the foetus to PAHs may dysregulate lung development and result in respiratory symptoms early after birth [36]. Jedrychowski and co-workers have in several studies used the birth cohort from Krakow [42–44] and measured the exposures to PAHs in pregnant women using personal air monitors, thus including both indoor and outdoor PAH exposure. In these studies, the prenatal PAH-exposure was significantly associated with respiratory infections, cough and wheezing days in infants and children during their first years of life.

Using a cohort from New York City, epigenetic markers associated with trans-placental PAH exposure and childhood asthma risk were investigated [45]. Methylation of a specific gene (ACSL3) in umbilical cord white blood cells was significantly associated with maternal airborne PAH exposure above 2.4 ng/m^3^. Parents reported asthma symptoms in children prior to 5 years of age. The authors suggested that this epigenetic change could function as a surrogate endpoint for trans-placental PAH exposure and/or a potential biomarker for environmentally-related asthma. In a study by Perzanowski and co-workers [46], pregnant women living in New York City were equipped with personal air samplers for measurement of 8 non-volatile PAHs and the semi-volatile PAH, pyrene. Prenatal exposure to cockroach allergen was associated with a greater risk of allergic sensitization. This risk was increased by exposure to non-volatile PAHs. Children lacking a common glutathione-S-transferase μ 1 (GSTM1) polymorphism appeared to be particularly vulnerable [46], indicating that GSTM1 may protect against development of sensitization by detoxifying PAHs.

In contrast to many of the original studies mentioned above a systematic review and meta-analysis of studies on the impact of prenatal exposure to air pollution on childhood wheezing and asthma, did not reveal statistically significant associations with PAHs [47]. It should be noted that the meta-analyses of PAHs’ association to childhood wheezing and asthma were based on only 7 studies, and the authors emphasized that further studies are needed to clarify effects of the individual compounds.

Also postnatal exposure to PAHs in early life has been reported to affect respiratory functions in children, possibly involving both separate and joint effects of trans-placental and postnatal PAH exposure [42, 43, 48]. In the Krakow birth cohort, prenatal PAH exposure was measured by personal monitoring of pregnant mothers, while postnatal exposure of the children was based on indoor and outdoor residential air levels of PAHs (both particle-bound and gaseous) and particle mass. Reduction of lung function parameters was associated both with prenatal PAH exposure and postnatal residential indoor PAH levels [48]. Findings in a cohort of pregnant women in New York City suggested that an interaction between prenatal exposure to PAHs and postnatal exposure to environmental tobacco smoke lead to increased respiratory symptoms and likelihood of diagnosed asthma in children aged 12 and 24 months [49]. The same research group also investigated the combined effect of prenatal PAHs and environmental tobacco smoke exposure and found an association with asthma, but not with the IgE levels at age 5–6 years [50]. Furthermore, both prenatal and postnatal exposure to pyrene and 8 non-volatile PAHs were measured in a longitudinal birth cohort in New York City using personal air monitors [51]. The results showed that early repeated exposure to pyrene during pregnancy and at age 5 to 6 was associated with non-atopic asthma among the young urban children. No apparent associations were observed with atopic asthma or non-volatile PAHs, including B[*a*]P. Overall, the studies on developmental effects from pre- and postnatal exposure to PAHs provides support for an association with asthma development in children.

*Childhood asthma.* Recent studies have found that exposure to PAHs is associated with onset, prevalence, and increase of asthmatic symptoms in children [36]. In a case–control study of 195 children strong associations between serum PAHs and biomarkers of childhood asthma were detected [52]. Data in this study indicated the potential involvement of naphthalene, 4H-cyclopenta[def]phenanthrene, 1,2-benzanthracene, chrysene and benzo(*e*)acephenanthrylene in childhood asthma. The levels of these PAHs were correlated with asthma-related biomarkers including immunoglobulin E (IgE), resistin, granulocyte–macrophage colony-stimulating factor (GMCSF) and interferon gamma (IFN-γ) as well as the cytokines; interleukin (IL)-4, IL-5, IL-8 (CXCL8) and IL-10. In another case–control study of 453 kindergarten children, exposure to PAHs measured in urine as 1-hydroxy-pyrene, was associated with asthma [53]. The effect of PAH exposure on asthma in children was suggested to be mediated by oxidative stress, as the exposure was also associated with 8-hydroxy-2′-deoxyguanosine (8-OHdG) in the urine, which is often used as a marker of oxidative stress. In a hospital-based case control study, cases of children with bronchial asthma (aged 1–14 years) were matched with healthy controls. Higher blood levels of phenanthrene and indications of oxidative stress (reduced blood level of glutathione and elevated malondialdehyde) were found in the group with bronchial asthma when compared to the matched controls [54]. Associations between increased urinary non-volatile metabolites of PAHs in children and possible allergic sensitization to mouse allergens (anti-mouse IgE levels) have also been reported [55]. However, the levels of PAH-metabolites were not associated with other respiratory symptoms. A cohort study on 315 asthmatic children (aged 6–11 years), explored the impact of particle-bound 4-, 5- and 6-ring PAHs on wheeze. Each of the three types of PAH exposure was associated with increased wheeze in asthmatic children, with phenanthrene having the highest relative impact [56].

Jung and co-workers found that obese young children compared to non-obese children could be more likely to develop asthma in association with higher exposure to PAHs, and semi-volatile methylphenanthrenes in particular [57]. The PAHs in these studies were measured by personal air monitors. The influence of aerosolized pollutants on the development of immune dysfunction in asthmatics has been suggested to be mediated through epigenetic remodelling. Several studies have reported that the epigenetic modifications associated with asthma have been found after pre- or post-natal PAH exposure [58]. Overall the majority of studies reviewed found an association between PAH exposure and childhood asthma. Importantly, the reported associations were not limited to B[*a*]P, but also encompassed low-molecular weight PAHs such as naphthalene, phenanthrene, and pyrene.

*Respiratory function and symptoms in children.* Exposure to air pollution has been reported to cause changes in respiratory functions, which also may be of importance for development and exacerbation of asthma [59]. However, few studies have examined the relationship between ambient PAHs and pulmonary function in children. In a cross-sectional study an association between individual exposures to the sum of PAHs with four, five or six rings and pulmonary function was reported in children (aged 9 to 18) without asthma, but no association was observed among asthmatic children of the same age [60]. An inverse association between biomarkers of PAH exposure and lung function was also seen among Mexican schoolchildren, in addition to decrease in pH of exhaled breath condensate, as a marker of airway inflammation [61]. In a panel study of children with asthma living in proximity to industry in Canada, exposure to different air pollutants including PAHs was measured using personal monitors. A small decrease in respiratory function was associated with an increase in the total concentrations of PAHs [62]. No associations between PAH metabolites in the urine and respiratory symptoms in 5 years old children were found in an inner-city birth cohort in New York City [55]. Thus, the epidemiological evidence for effects of PAH exposure on respiratory function in children is so far inconclusive.

*Adult asthma.* Epidemiological studies suggest that exposure to air pollution affects asthma also in adults and the elderly, but few studies have examined the impact of PAH exposure on adult asthma. In a recent case–control study, Huang and colleagues explored the quantitative relationship between urinary PAH-metabolites and asthma [63]. The concentrations of 12 urinary PAH metabolites were measured and the potential associations with adult asthma were analysed by logistic regression. The authors reported that ∑OH-PAHs were significantly associated with elevated risk of adult asthma with odds ratios of 1.34. Interestingly, the odd ratios for adult asthma were higher for some of the low-molecular weight PAH-metabolites; being 2.04, 2.04, 2.38, 2.35, for 2-hydroxy-fluorene, 1-, 4-hydroxy-phenanthrene, 1-hydroxy-pyrene, respectively. Again, this indicates an association between low-molecular weight PAHs and asthma also in adults.

*Respiratory function and symptoms in adults*. Changes in respiratory functions in adults induced by air pollution have been linked to development and/or exacerbation of asthma and COPD [64]. Within a representative sample of the Canadian population (6–79 years) changes in urinary metabolites of eight PAHs (2-hydroxy-napthalene, 1-, and 2-hydroxy-phenanthrene, 2-, 3-, and 9-hydroxy-fluorene and 3- and 4-hydroxy-phenanthrene) were associated with significant decrements in lung function [65]. A study of elderly in Korea, exposure of PAHs measured as the pyrene metabolite 1-hydroxy-pyrene in the urine was associated with compromised lung function in the elderly, and haplotype-based CYP1A1 polymorphisms were suggested to modify the risk [66]. Furthermore, in a Chinese population, a relationship between increased levels of urinary PAH metabolites and reduced lung function was reported [67]. In another study of the Chinese population, urinary hydroxy-PAHs levels were marginally negatively related to lung function in all participants [68]. Ambient PAH-exposure has also been associated with cough prevalence in a study of patients with chronic cough. The association appeared to be stronger in non-asthma patients. However, the authors emphasize that the study did not account for other co-pollutants, and that further studies on a larger sample size would be needed [69].

Associations between increased levels of urinary PAH metabolites and decreases in lung function parameters have also been observed in occupational settings. In a longitudinal investigation involving more than 1000 coke-oven workers with follow-up periods from 2010 to 2014, urinary concentrations of 12 PAH metabolites were measured [70]. Several of the metabolites (1-/2-hydroxy-naphthalene, 2-/9-hydroxy-fluorene, 1-/2-hydroxy-phenanthrene, 1-hydroxy-pyrene and the sum of hydroxy-PAHs) were significantly associated with accelerated decline in lung function over a 4-years follow up. Similarly, an association between decreases of a lung function parameter and increasing total level of six PAHs urinary metabolites (1-/2-hydroxy-naphthalene, 2-hydroxy-fluorene, 2-/9-hydroxy-phenanthrene, 1-hydroxy-pyrene) was observed in a study of diesel engine testing workers [71].

Thus, the majority of studies reviewed seems to suggest an association between PAH exposure, measured as urinary PAH metabolites, with respiratory functions and symptoms in adults. This is shown both in environmental and occupational settings. Moreover, the reported associations also encompassed low-molecular weight PAHs.

### Respiratory mortality in adults

Air pollution from both ambient and occupational sources has been shown to exacerbate and increase mortality from obstructive lung diseases [4]. Whether PAHs are associated with increases in respiratory mortality is more uncertain. A historical cohort of asphalt workers from different countries, including nearly 60,000 men, has been used to elucidate this. In a study using this large cohort, it was concluded that exposure to PAHs might have contributed to mortality from obstructive lung diseases among the workers [72]. However, confounding and bias could not be fully ruled out as an explanation for the observed associations. Hence it remains unclear whether ambient PAH exposure may be a contributing factor to the respiratory mortality from air pollution.

### Respiratory infections

Epidemiological studies have reported associations between exposure to air pollution and increased risk for respiratory infections [73–75], and people with infections seem to be more susceptible to health effects induced by air pollution [76, 77]. Less is known regarding specific components involved. In a study from Lodz in Poland, the urine concentrations of 1-hydroxy-pyrene were analysed in both mothers and the children during the first two years of life. The results suggested that PAH-exposure of mothers was a risk factor for airway infection in children, while PAH-exposure of children was a risk factor for food allergy [78]. Thus, supporting a previous study of a cohort with 333 new-borns in Krakow reporting that PAHs measured by personal air monitoring of mothers increased the susceptibility of new-borns and young infants to respiratory infections [42]. The 30-day average of ambient PAHs (a total of twelve PAHs measured in both the gas- and particle phases) has also been associated with early-life susceptibility to bronchitis [79]. Overall, these limited studies suggest that a causal role of PAHs in the increased risk of respiratory infections cannot be excluded.

### Evaluation of the epidemiological studies

Few epidemiologic studies have elucidated any possible association between air-borne PAH exposure and non-malignant respiratory diseases. Furthermore, in all studies the specific role of PAHs is difficult to disentangle from other combustion-related pollutants of potential impact on the respiratory diseases. Thus, the epidemiological studies so far reported needs to be interpreted with caution. Nevertheless, the studies combined support the notion that air-borne PAH exposure may contribute to development and/or exacerbation of respiratory disease and dysfunction. The strongest evidence is currently for an association between PAHs and asthma development and respiratory function in children. This is supported by studies on both pre- and post-natal exposure as well as exposure during childhood. Exposure to PAHs in adults seems also to be linked to respiratory function and symptoms. For other respiratory outcomes, there are indications of associations with PAH exposure, but further studies will be needed to confirm this. Furthermore, the available epidemiological evidence suggests that in particular low-molecular weight (two-four rings) PAHs may be associated with non-malignant pulmonary effects. However, due to the prevalent use of pyrene and naphthalene metabolites as biomarkers of airborne PAH-exposure, as well as larger impact of foodborne exposure on higher molecular weight PAH-metabolite levels, the associations between specific PAH-species and pulmonary outcomes should be interpreted with caution. Importantly, as many constituents of air pollution derive from identical sources and processes (i.e. combustion processes), they are almost impossible to disentangle sufficiently in epidemiological studies. In accordance with the Bradford Hill criteria for causation in epidemiological evidence [80], our focus in the next section is whether there are plausible biological mechanisms that could explain the epidemiological observations and whether there is coherence between epidemiological evidence and experimental laboratory findings.

## PAH exposure and respiratory outcomes: do experimental studies support causal inference?

The mechanistic understanding of PAH-toxicity is largely based on studies of biological processes linked to carcinogenesis. Chemical properties important for mechanisms involved in cancer development may also enhance the risk for development or exacerbation of asthma and COPD and acute and recurring respiratory infections. However, it has become increasingly clear that PAHs affect several signaling pathways that may not be directly related to their carcinogenic potential. The relative significance of the different effects induced by PAH-exposure, likely varies depending on the health outcome in question.

**Fig. 1:**
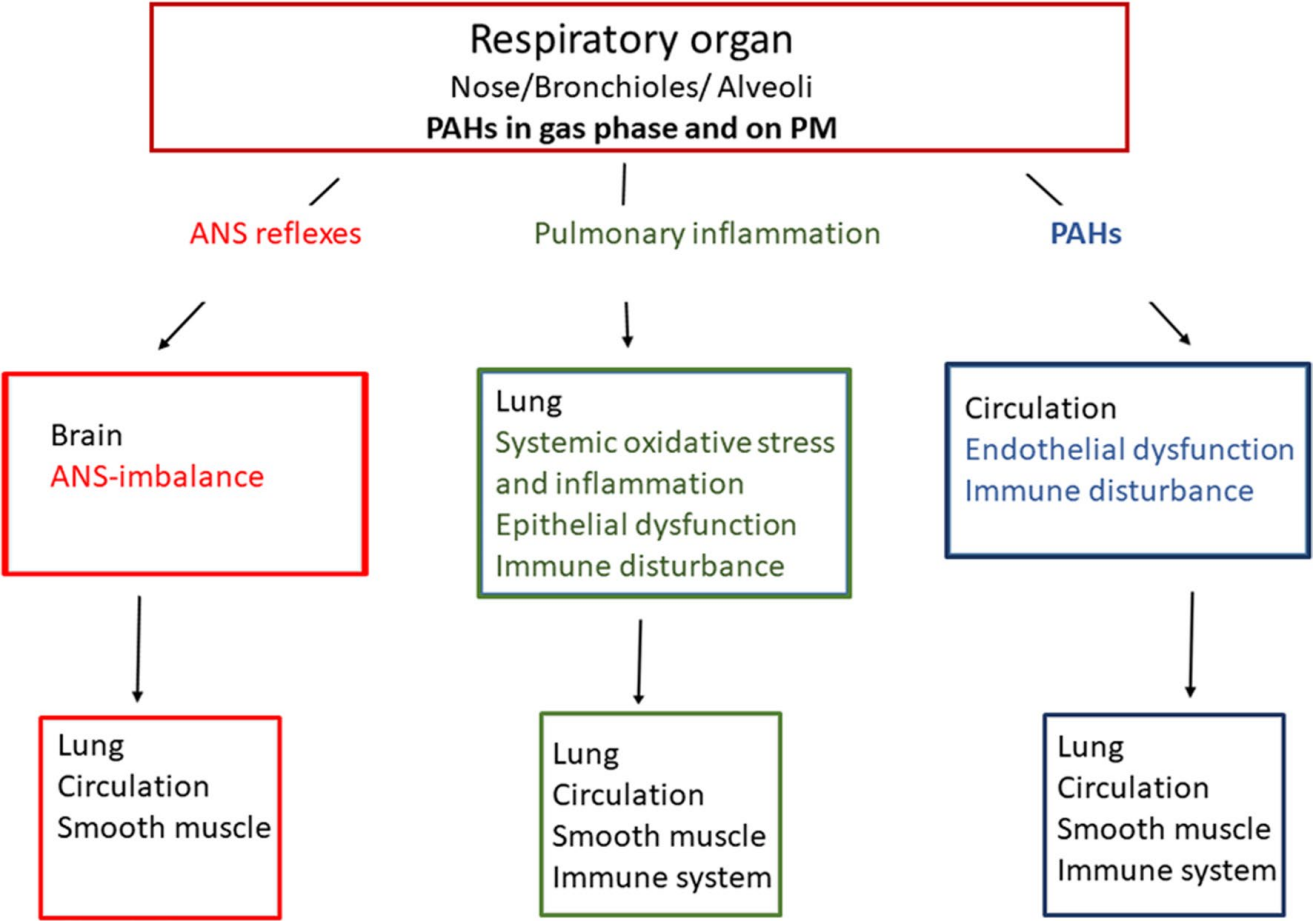
Possible mechanisms linking polycyclic aromatic hydrocarbons (PAHs) with non-malignant respiratory diseases. Three general lines of causality are suggested: (i) Distortion of autonomic nerve endings in the lungs causing loss of vascular and smooth muscle control reflexes via the autonomic nervous system (ANS; red), (ii) Pulmonary inflammation (green) and (iii) direct effects of detached PAHs from particles or PAHs from gas phase affecting lung/circulation directly (blue)

PAHs on combustion PM and in the gas phase may in principle contribute to development of non-malignant respiratory diseases via three main physiological mechanisms (Fig. 1). A well-known mechanism is induction of local pulmonary inflammation (ii). Exposure of pulmonary macrophages, epithelial and local endothelial cells to PAHs may trigger the release of pro-inflammatory mediators into the circulation [7]. This may give systemic inflammatory responses, which can amplify the pulmonary response. It has also been suggested that inhaled combustion PM/PAHs may trigger receptors in the autonomic nervous system of the respiratory tract [81], thereby triggering an autonomic nerve system (ANS)-reflex (i). This may include bronchiolar muscle contraction and inflammation via tachykinin release. Such changes may also enhance ongoing inflammation and thus be directly linked to exacerbation of asthma [82]. Furthermore, as a third possibility PAHs detached from PM and PAHs in the gas phase may cross the epithelial barrier (iii) and react with essential components in the blood or cells of more distantly located tissues [20, 83, 84].

Both genetic and environmental factors are important for non-malignant respiratory diseases [85]. Dysregulated lung development and asthma may be triggered by exposure occurring prenatally, in early post-natal life as well as in adult life [36]. Chemical-induced effects on cellular migration and differentiation may impair lung development [86], while effects on the immune system may increase the risk of asthma development and respiratory infections [73, 75, 87–89]. COPD, a progressive lung disease characterized by chronic bronchitis and emphysema, is mostly triggered by irritating cellular events occurring during adult life [90].

**Fig. 2:**
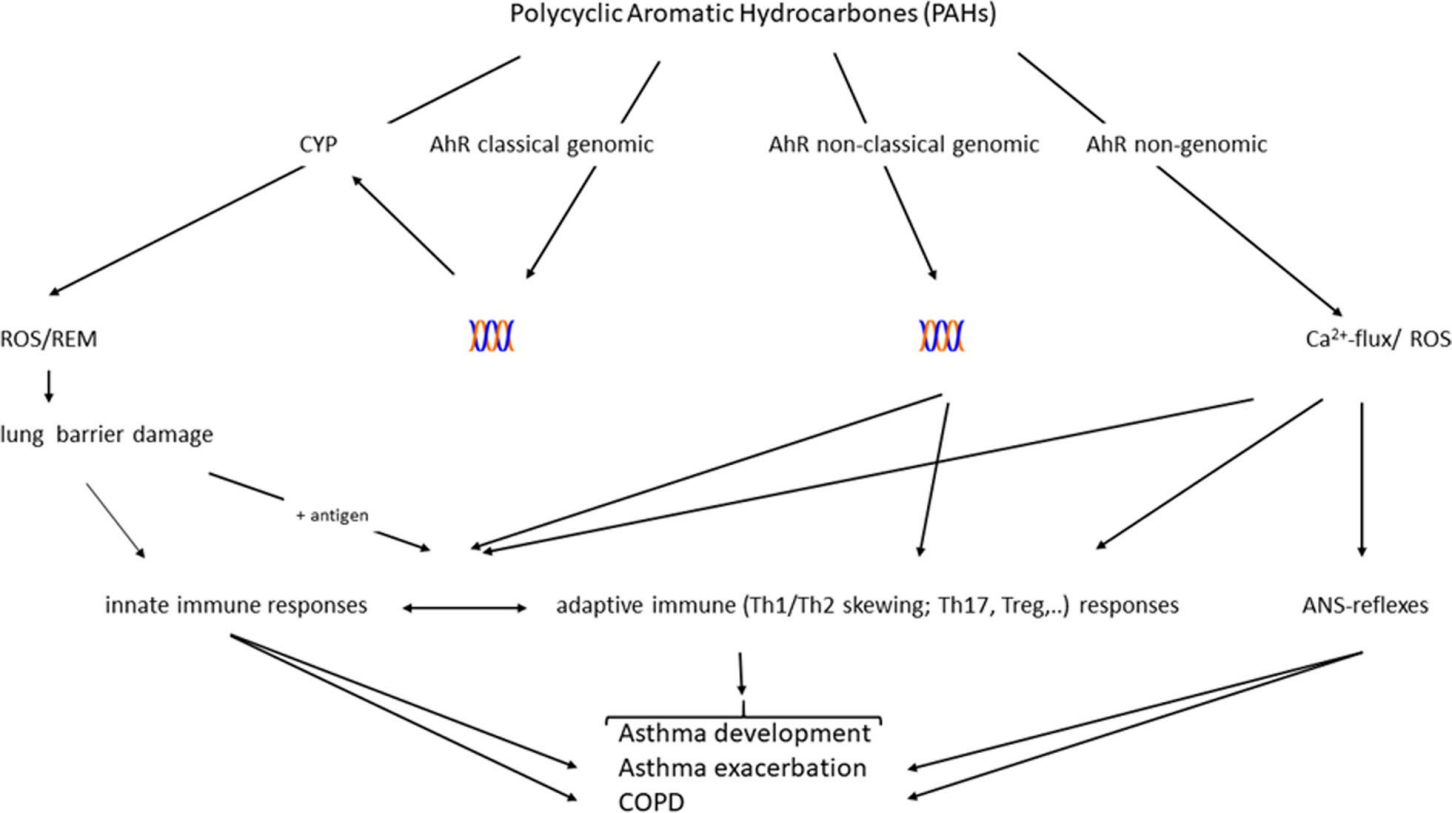
Possible mechanisms linking polycyclic aromatic hydrocarbons (PAHs) with non-malignant respiratory diseases via aryl hydrocarbon receptor (AhR). Four general lines of causality are suggested: (i) formation of reactive oxygen species (ROS) and reactive electrophilic metabolites (REM) and damage of epithelial/endothelial lung barrier; triggering of (ii) AhR classical genomic, iii) AhR non-classical genomic or iv) AhR non-genomic pathway; DNA (  ); Cytochrome P450 enzymes (CYP); autonomic nervous system (ANS); chronic obstructive pulmonary disease (COPD)

Central physiological- and pathological responses involved in non-malignant respiratory diseases, include redox imbalance, oxidative stress, inflammation, bronchial muscle constriction, epithelial- and endothelial dysfunction and dysregulated lung development. Notably, combustion particles and/or PAHs exposure have been shown to activate such responses [7, 8, 31]. PAHs may bind and activate cellular receptors including the AhR [29–31] and the G protein-coupled receptor (GPCR) β_2_-adrenergic receptor (β_2_-AR) [91, 92]. Furthermore, PAH metabolites and ROS generated as by-products of PAH-metabolism may interact directly with ion transporters and enzymes [32]. This may lead to activation of a variety of signaling pathways and target genes in cells central for such physiological- and pathological processes. A more detailed list of these possible molecular triggering events for PAHs’ biological responses are included in Table 2, and suggested mechanistic links are presented in Fig. 2.

**Table 2:** Molecular effects of PAHs induced by the parent compounds, reactive oxygen species (ROS) and electrophilic metabolites

Cellular induced ROS formation/oxidative stress
Redox cycling of eg PAH-metabolites
Activation of membrane bound oxidases
Damage to mitochondria
Activation of ROS defense (macrophages)
Membrane receptors, transporters and channels e.g. β_2_-AR
Ca^2+^ signaling
cAMP signaling
Phosphorylation signalling (including inhibition of phosphatases)
Intracellular receptors including aryl hydrocarbon receptor (AhR)
Classical genomic: CYP enzymes
Non-classical genomic: cross talk with e.g. NF-κB
Non-genomic signalling:
Protein phosphorylation via kinases (ScC)
Membrane order/caveola Ca^2+^ signaling
Genotoxic effects
DNA damage and epigenetic changes

### PAHs and reactions with cellular receptors linked to respiratory diseases

PAHs in gas phase or detached from particles may cross epithelial barrier and reach more distantly located cells [20, 83, 84]. Thus, PAHs may easily react with intracellular receptors including AhR as well as plasma membrane-bound receptors on endothelial cells, immune cells, vagus nerve or even possibly also more distally located smooth muscle that surround the bronchioles. Of interest, particle-bound PAHs depositing in the bronchi and bronchioles appear mostly to act locally in the lung tissue where the majority is also metabolized by lung cells, while deposition in the alveolar region leads to rapid translocation of unmetabolized PAHs into circulation [84].

*Aryl hydrocarbon receptor (AhR)* is an intracellular receptor playing a central role in modulating toxicity of PAHs and other environmental pollutants with aromatic structures. In its classical genomic mode of action, ligand-activated AhR dimerizes with the AhR nuclear translocator (ARNT) and binds to so-called xenobiotic response elements (XREs) in the promotor region of genes coding for e.g. the cytochrome P450 (CYP) enzymes CYP1A1/CYP1B1 [20, 29, 30, 93, 94]. Metabolism of PAHs by CYP-enzymes may form ROS and reactive electrophilic metabolites, which in addition to damaging DNA, also may inactivate important signaling enzymes and transporters by oxidizing sulfhydryl groups and trigger inflammation [20, 93].

More recently, it has become clear that AhR elicits a number of other cellular functions, and plays important roles in immune regulation [95, 96]. Certain chemicals binding to AhR may also mediate inflammatory signals via non-classical pathways. The non-classical genomic pathway includes cross talk with the nuclear factor-κB (NF-κB) family of transcription factors as well as other transcription factors independently of ARNT [97, 98]. The AhR-ligand binding is also suggested to mediate immune modulating effects via so-called non-genomic signaling, where AhR acts as a cytosolic signaling protein and not as a nuclear transcription factor. These non-genomic pathways may in addition include effects on estrogen receptor (ER)-α and Src, as well as increases in intracellular Ca^2+^ [99, 100].

The AhR is strongly expressed in immune cells and in structural parts of the lungs, including epithelial layer, submucosal glands, fibroblasts and endothelial layer of the pulmonary vasculature [95, 96]. Both adaptive and innate immune cells require AhR-signaling at critical checkpoints. It is well recognised that activation of AhR by ligands may suppress T cell-dependent immune responses and influence the balance of Th17 versus regulatory T-cells [30] as well as dendritic cell function [101]. Furthermore, it has been found that exposure to AhR-ligands including B[*a*]P, resulted in enhanced calcium- and ROS-dependent mast cell signaling, leading to degranulation, mediator and cytokine release, as well as the in vivo anaphylactic response [102].

The AhR exhibits immune-modulating properties that may fine-tune respiratory immune responses. However, a duality in effects of AhR and its ligands on pulmonary inflammation currently prevents simple conclusions [103]. For instance, AhR-activation by the classical ligand, 3, 4, 7, 8-tetrachlorodibenzo-p-dioxin (TCDD) appears to suppress quartz-induced inflammation in silicosis [104]. Other studies suggest that AhR-activation in immune and non-immune cells in general may contribute to development and progression of COPD as well as asthma [30]. Differences in the ligands with regard to metabolism and induced toxicity, binding to AhR and subsequent activation of classic or non-classic genomic- or non-genomic pathways, illustrate that the role of AhR in respiratory diseases is multifaceted [103, 105].

The ability of PAHs to activate the classical genomic AhR-pathway, has often been considered to reflect their ability to bind and activate AhR in general. However, the relationship between ligand binding, AhR-activation, and downstream signaling appears to be more complex than previously considered. Recent data suggest that pyrene may bind to AhR with comparable affinity as B[*a*]P without inducing CYP1-expression, but activating AhR-dependent Ca^2+^-signaling instead [105]. Conceivably, binding of different AhR-ligands may lead to differences in the receptor conformation resulting in different activation of downstream signaling. Thus, some PAHs that are not classified as AhR-activators due to limited or no effects on CYP1-expression, may have other effects through interaction with AhR.

*β*_*2*_*-adrenergic receptor (β*_*2*_*-AR)* belongs to the seven-transmembrane G-coupled receptors and binds epinephrine and norepinephrine (adrenaline and noradrenaline). Activation of β_2_-AR in the airways initiates smooth muscle relaxation and bronchodilation, in part through activation of intracellular calcium and cyclic AMP (cAMP) signaling and is therefore among the key therapeutic targets for treatment of obstructive pulmonary disorders [106]. For decades, β-agonists have been widely used for bronchodilation in asthma and COPD. Notably, a PAH-mixture with more than 50% pyrene impaired β_2_-AR-function in airway epithelial and smooth muscle cells, and interfered with β-agonist (procaterol) treatment [107]. This effect was associated with down-regulation of β_2_-AR expression levels in the cells. In a follow-up study, it was shown that prenatal exposure of mice to the same PAH-mixture reduced β_2_-AR expression levels measured at day 42 after birth [108]. Moreover, postnatal PAH exposure was borderline associated with increased airway hyper-reactivity in wildtype mice (*P* = 0.055, two-tailed *t*-test). In contrast, no indications of effects were seen in β_2_-AR knockouts, thus supporting the hypothesized role of the β-adrenergic system in the pulmonary effects of PAHs [108]. These studies, complement earlier findings by Irragaray and colleagues, showing that B[*a*]P impaired epinephrine-induced lipolysis in adipocytes through adrenergic receptors and caused weight-gain in mice at relatively low doses compared to what is typically used to induce tumorigenic responses [109]. Studies in human endothelial and embryonic kidney cells (HMEC-1 and HEK293) have shown that B[*a*]P may interact directly with the ligand binding pocket of the β_2_-AR at low concentrations (Kd ~10 nM), leading to a subsequent increase in intracellular Ca^2+^ and cAMP, and desensitization of β_2_-AR [91, 92, 110]. These studies illustrate that PAHs may interfere with adrenergic signaling in the airways. Although the full implications of the β_2_-AR interaction still remain unclear, the current studies suggest that PAHs could desensitize adrenergic receptor function and thus interfere with epinephrine/norepinephrine signaling. Such effects could potentially prevent bronchodilation and possibly contribute to development or exacerbation of obstructive disorders including asthma and COPD.

### Pulmonary oxidative stress and inflammation in respiratory diseases

Oxidative stress and inflammation are considered central in both asthma and obstructive lung diseases. Regulation of protein phosphatases and intracellular antioxidant molecules such as nuclear factor erythroid 2-related factor 2 (NRF2), glutathione and peroxidase system are suggested to be involved [111]. Oxidative stress is suggested to have a potent effect on Th1/Th2-skewing of the immune response and has an important role in asthma pathogenesis [112]. AhR-ligands including PAHs have been reported to increase oxidative stress pathways [95, 113]. PAHs are metabolically activated by cytochrome P450 forming reactive species including epoxides, peroxides as well as semiquinones and quinones, which next also can generate ROS. The increased ROS may also be a result of secondary mitochondrial damage. Such reactive metabolites, may trigger both pro-inflammatory and anti-inflammatory signaling pathways, leading to transcriptional upregulation of genes involved in regulating immune responses [12, 36, 111, 114]. Furthermore, a disturbed red/ox balance may lead to accumulation of unfolded proteins and result in an “unfolded protein response” which intersects with different inflammatory and stress signaling pathways [115].

B[*a*]P exposure has been reported to induce CXCL8 by an AhR-dependent pathway in primary human macrophages [116]. In epithelial lung cells, B[*a*]P and PAHs like pyrene and 1-nitropyrene are reported to induce expression of CXCL8 and other chemokines/cytokines [117–119]. It should be noted that AhR appears to exhibit both pro- and anti-inflammatory properties [7]. In accordance with this, B[*a*]P attenuated pro-inflammatory phenotype of macrophages by inducing anti-inflammatory IL-10 expression in an AhR-dependent manner [120]. Further studies in vitro on lung epithelial cells suggest that 1-nitropyrene may induce Ca^2+^-signaling at least partly through activation of β_2_-AR, and suggest that both β_2_-AR and Ca^2+^-signaling may be involved in induced CXCL8 responses [121]. However, the concentrations required for 1-nitropyrene to induce Ca^2+^, and the various PAHs to induce cytokine responses in vitro, appear to be several orders of magnitude higher than what would be reached under real-life exposure scenarios.

A study with DEPs showed that both native DEPs and a corresponding methanol DEP-extract, but not residual (washed) DEPs, induced marked expression of the inflammatory mediators cyclooxygenase (COX-2), IL-6 and CXCL8, as well as cytotoxicity, in human bronchial epithelial cells. The analysis of the DEP-extracts indicated that most of the analysed PAHs and PAH-derivatives were extracted from the particles [122]. Several other studies suggest that the majority of pro-inflammatory effects of DEP could be attributed to nonpolar-soluble components [123–125]. Moreover, nonpolar-soluble DEP-extracts, pyrene, and pyrene derivatives which induce AhR non-genomic Ca^2+^-signaling [105] exacerbated cytokine responses induced by the Toll-like receptor 3 ligand poly I:C, at concentrations where extracts and PAHs alone were unable to stimulate cytokine release [125, 126].

The above studies show that there are several mechanisms by which PAHs may induce or exacerbate inflammatory responses in cell types linked to the airways, and thus contribute to both exacerbation and development of lung diseases [36].

### Immunological responses involved in allergic asthma

Some epidemiological studies have indicated an association between PAH exposure and allergic asthma [46, 55]. PAHs may also act through IgE to stimulate inflammatory responses, enhance allergic reactions to induce respiratory diseases [36, 127]. Most interestingly, experimental studies indicate that pyrene enhances allergic IgE responses in mice [128], thus further supporting the notion that AhR non-genomic Ca^2+^-signaling may be involved in several processes important for asthma. Exposure to PAHs in vivo may also influence B cell and T-helper cell differentiation by skewing immune responses towards a Th2-specific profile, which favours B-cell production of IgE and eosinophils, hallmarks of allergic inflammation and allergic asthma [36, 112]. Low-dose B[*a*]P in mice was suggested to enhance allergic airway inflammation by facilitating Th2 responses and activating lymph node cells. However, a high B[*a*]P dose might contribute in activating both Th1 and Th2 responses [129]. Intranasal exposure of mice to another environmental PAH, indeno[1,2,3-cd]pyrene, significantly enhanced antigen-induced allergic inflammation, which was absent in dendritic cell-specific AhR-null mice [130].

Primary bronchial epithelial cells exposed to DEP showed upregulation of IL-33, IL-25, and epithelium-derived thymic stromal lymphopoietin (TSLP). These effects were linked to AhR binding, implying a possible role of PAHs [131]. Bronchial biopsies from patients with allergic severe asthma were included in the same study and showed that high AhR nuclear translocation was associated with higher bronchial gene and protein expression of IL-33, IL-25, and TSLP [131]. Furthermore, findings from a recent study using a mouse model of asthma suggested that B[*a*]P facilitates allergen (Der f 1)-induced epithelial cytokine release through the AhR-ROS axis via TSLP and IL-33 [132]. Increased expression of AhR is also suggested to play a role in the pathogenesis of allergic rhinitis, contributing to chemokine production in nasal mucosa [133]. Other studies indicate that PAHs can act directly on AhR in T cells, leading to enhanced Th17 differentiation [134]. These cells, as a source of IL-17 and IL-22, are implicated in the pathogenesis of asthma [36, 135, 136]. Using a combination of in vitro cell cultures and in vivo mouse models, ambient ultrafine particles were recently found to exacerbate allergic airway inflammation by promoting a Jagged 1-Notch 4-dependent interaction between lung alveolar macrophages and allergen-specific T cells, leading to augmented Th cell differentiation [137]. It is interesting to note that Manners and co-workers have developed a mouse model linking maternal exposure to DEPs with asthma susceptibility in offspring [114]. They found that the development of asthma was dependent on natural killer cells and associated with increased transcription from AhR- and oxidative stress-regulated genes.

The studies described indicate several mechanisms by which PAHs may induce or exacerbate immunological responses involved in allergic reactions, and thus contribute to both exacerbation and development of asthma.

### Respiratory neuronal reflexes and respiratory symptoms

Respiratory reflexes are responsible for symptoms such as cough and bronchospasm and are regulated by vagal afferent nerves/C-fibers, which innervate the airways. In a recent study, Robinson and co-workers [81] showed that DEP (SRM-2975) activated the C-fibers in guinea pigs. The organic extract and not the cleaned particles evoked depolarization of vagus nerves isolated from guinea pig and human. Most interestingly, this effect was also seen after exposure to very low concentrations (1 nM) of phenantrene, which is among the most dominating PAHs in urban air [20]. The changes in depolarization were inhibited by the AhR-antagonist CH223191 and by the transient receptor potential (TRP) channel-antagonist ankyrin-1, strongly suggesting that the mechanisms for these effects were depending on AhR-activation that resulted in subsequent influx of calcium through TRP channels [81].

### Lung epithelial barrier and respiratory infections

Experimental studies provide support for a role of PAHs in increased susceptibility to virus and bacteria when exposed to air pollution. Possible mechanistic explanations could be due to factors like; disrupted lung epithelial barrier, reduced mucociliary clearance, immune defects including reduced pathogen recognition and killing, impaired cytokine release and surface receptor expression to pathogen products, Th2-skewed response, impaired clearance of infectious agents and delayed recovery from severe disease [73, 75]. The mechanisms would thus include both innate and adaptive immune responses.

Although still far from fully elucidated, cytotoxicity from reactive PAH metabolites and ROS may damage lung epithelial cells and thereby disturb essential barrier-functions. Alternatively, PAHs may disrupt the epithelial barrier by affecting tight junctions and/or gap junctions via AhR-dependent mechanisms [105, 138]. If these effects of PAHs are sufficiently strong, this could lead to secondary pro-inflammatory reactions with further amplification of the epithelial damage [139].

As AhR has an important function linked to development and differentiation of the immune system, long-term exposure to ligands may also affect the vulnerability to respiratory infections. Notably, M1/M2 macrophage polarization [140–143], Th1/Th2 polarization [144, 145], dendritic and mast cell function [88, 102] seem to be linked to non-classical AhR genomic or AhR non-genomic pathways. In addition to immune cells locally in the lung or more distant, both epithelial and endothelial cells are targets of AhR-mediated changes linked to immune responses [146]. Experimental data from mouse models highlight that the AhR via non-classical genomic and/or non-genomic pathways may be involved in respiratory effects [95]. Interestingly, in RAW264.7 macrophages stimulated by bacterial endotoxins like lipopolysaccharides (LPS), the transcription of IL-6 and TNF-α was suppressed by low-molecular-weight PAHs like fluoranthrene and 1-nitropyrene [147]. Furthermore, 1-nitropyrene was also found to reduce phagocytosis.

Whether interactions between PAHs and the AhR may have implications for respiratory infections is difficult to predict due to the many-faceted role of AhR, and may be highly dependent on the experimental model. Accordingly, both enhancement and protection against infections have been demonstrated after exposure to AhR-ligands [146, 148, 149]. Activation of AhR by TCDD in developing mice might contribute to modified responses such as greater bronchopulmonary inflammation and activated virus-specific CD4^+^T cells, after exposure to respiratory influenza viral infection as adults [150]. Furthermore, activation of AhR in adult mice is reported to increase pulmonary neutrophilia, but diminish host resistance to influenza A virus [151]. In addition, TCDD has been shown to protect against S. pneumoniae-induced mortality and reduce pulmonary bacteria burden in mice [149].

Many of the examples above are from studies on AhR knock-down/ TCDD-ligand binding. However, as low molecular weight PAHs also seem to bind to AhR and in part trigger non-classical signaling or interfere with AhR-functions [81, 105, 147], it is tempting to speculate that also these mechanisms may affect host susceptibility and responses to respiratory infections [147].

### Comments to mechanistic studies

The published literature on how PAHs may affect cellular processes of central importance for development and exacerbation of non-malignant respiratory diseases is extensive. A full-scale systematic review of the experimental literature would therefore have been too comprehensive compared to the purpose of the present paper, which was to review the epidemiological evidence and provide some hypothesis or suggestions for potential mechanisms that could explain associations observed in humans. The mechanistic studies discussed in this section thus rather highlight selected findings of potential importance to understand the epidemiological evidence, and should be interpreted as such.

Several other well-known and important genetic and environmental factors should also be taken into consideration when extrapolating knowledge from animal studies and experimental studies in vitro to human. Most importantly, much of the work described is done with single PAHs, thus the concentrations used may be substantially higher than realistic real-life exposure. On the other hand, in a human situation cells are continuously exposed to a complex mixture of PAHs from various sources. Furthermore, there are studies indicating that DEP-extracts may give AhR-mediated effects even at extremely low concentrations of PAHs [152, 153].

## Conclusions

Human epidemiological studies combined with experimental studies give support to the hypothesis that exposure to air-borne PAHs may contribute to the enhanced risk of non-malignant respiratory diseases associated with air pollution exposure.

Epidemiological studies show strongest evidence for an association between PAHs and asthma development and respiratory function in children. This is supported by studies on both prenatal and postnatal exposure as well as exposure during childhood. Exposure to PAHs in adults seems also to be linked to respiratory function and symptoms, exacerbation of asthma and increased morbidity/mortality of obstructive lung diseases. However, the potential roles of PAHs are difficult to disentangle from other components originating from combustion processes, including the combustion particles as such.

Experimental studies in vivo and in vitro, give support to the epidemiological associations by providing possible mechanisms linking PAH exposure to various non-malignant respiratory diseases. Effects of PAHs in respiratory tissue or cells may partly be mediated by classical AhR-dependent mechanisms, including binding to AhR, increased gene expression and/or metabolic activation by CYP-enzymes. Such events have been found to induce pro-inflammatory mediators and oxidative stress. Also more subtle and less studied non-classical AhR mechanisms, such as alteration in calcium signaling and cell membrane depolarization may be involved in some of these effects. Other experiments suggest that certain PAHs may be important for immune-modulating properties. However, a number of uncertainties remain to be clarified including dose–response relationships, the mechanisms involved and the relative importance of different PAH species in such processes.

The reviewed literature indicates that effects of ambient air PAH exposure extend beyond B[*a*]P and lung cancer. A broader range of PAH-species may contribute to several of the adverse pulmonary outcomes associated with air pollution and ambient air PM-exposure. Thus, it is highly uncertain if the current regulatory guideline, which is based on B[*a*]P, sufficiently protects against the various non-malignant respiratory health outcomes discussed in this review. Further epidemiological and experimental studies are required to establish a clear causal link between exposure to B[*a*]P and other PAH species and non-malignant respiratory diseases.

## Data Availability

Not applicable.

## Abbreviations

ADRs: Adrenergic receptors
AhR: Aryl hydrocarbon receptor
ARNT: AhR nuclear translocator
B[*a*]P: Benzo[*a*]pyrene
β_2_-AR: Beta-2 adrenergic receptor
COPD: Chronic obstructive pulmonary disease
CYP: Cytochrome P450
[Ca^2^^+^]_*i*_: Cytosolic concentration of calcium
DEP: Diesel exhaust particles
ETS: Environmental tobacco smoke
FEV1: Forced expiratory volume in one second
FVC: Forced vital capacity
FEF_25-75_: Forced expiratory flow at 25–75% of FVC
GPCRs: G protein-coupled receptors
GSH: Gluthatione
IgE: Immunoglobulin E
IL-: Interleukin-
NRF2: Nuclear factor erythroid 2-related factor 2
NF-κB: Nuclear factor-κB
OR: Odds ratio
PM: Particular matter
PAHs: Polycyclic aromatic hydrocarbons
ROS: Reactive oxygen species
TCDD: 3, 4, 7, 8-Tetrachlorodibenzo-p-dioxin
TSLP: Thymic stromal lymphopoietin
TRP: Transient receptor potential
WHO: World Health Organization
